# The association between triglyceride-glucose index and cognitive function in nondiabetic elderly: NHANES 2011–2014

**DOI:** 10.1186/s12944-023-01959-0

**Published:** 2023-11-06

**Authors:** Baojian Wei, Qianni Dong, Jinlong Ma, Aihua Zhang

**Affiliations:** 1 School of Nursing, Shandong First Medical University & Shandong Academy of Medical Sciences, No.619 Changcheng Road, Daiyue District, Taian, 271000 China; 2 School of Nursing, Yanbian University, Yanji, China

**Keywords:** Cognition, Insulin resistance, NHANES, Nondiabetic elderly, Triglyceride glucose index

## Abstract

**Background:**

The relationship between Insulin resistance (IR) evaluated through homeostasis model assessment insulin resistance (HOMA-IR) and cognitive function is controversial among nondiabetic individuals. No study so far has reported the association between the IR evaluated through triglyceride glucose (TyG) index and cognitive function among nondiabetics. This study aims to assess this association among US nondiabetic older elderly.

**Methods:**

Data were obtained from the 2011–2014 National Health and Nutrition Examination Survey (NHANES). Low cognitive function was evaluated using the Consortium to Establish a Registry for Alzheimer’s Disease Battery for immediate word list learning (CERAD-WL) and delayed recall (CERAD-DR) test, the Animal Fluency Test (AFT), and the Digit Symbol Substitution Test (DSST). Logistic regression analyses were conducted to compute the odds ratio (OR) and 95% confidential interval (CI) to examine the association between the TyG index (continuous and quartiles) and low cognitive function.

**Results:**

A total of 661 nondiabetic older adults were included with a mean age of 68.62 ± 6.49 years. Compared to the 1st quartile of the TyG index, participants in the TyG index 4th quartile were associated with low cognitive function evaluated through the CERAD test (CERAD-WL and CERAD-DR) [OR: 2.62; 95% CI (1.31, 5.23); *P* < 0.05]. Subgroup analyses showed that females (OR_Q4 VS Q1_: 3.07; 95% CI (1.04, 9.05); *P* < 0.05) and smokers (OR _Q4 VS Q1_: 2.70; 95% CI (1.01, 7.26); *P* < 0.05) categories were related with a higher risk of low cognitive function.

**Conclusions:**

A high TyG index was strongly correlated with low cognitive function evaluated through the CERAD test (CERAD-WL and CERAD-DR) among US nondiabetic older women. The management of IR in women might be beneficial to primarily prevent low cognitive function among nondiabetic older elderly.

## Introduction

The global prevalence of age-associated cognitive impairment and dementia is projected to rise substantially [1]. Older adults have a higher risk for cognitive impairment, which is a prodromal stage of dementia [2]. With population aging, cognitive decline can easily progress to dementia, and become a public and economic burden [3]. Furthermore, although the Food and Drug Administration has recently approved two anti-amyloid therapies that may slow disease progression, no effective treatment for dementia exists. Since cognitive decline causes great distress to the patient’s quality of life [4–6], early detection of risk factors is crucial.

Diabetes, a metabolic disease, accelerates the progression from mild cognitive impairment to dementia [7]. Although the mechanism of cognitive impairment in patients with diabetes is uncertain, insulin resistance (IR) is known to be involved in the primary mechanism [8]. The severity of diabetes is closely related to the severity of IR and cognitive impairment [9], implying that more severe diabetes is usually associated with a higher IR index and lower cognitive performance [10]. In addition, higher IR and glucose intolerance are related to worse cognitive performance, even before diabetes [11–13]. IR and cognitive function are inversely correlated in the diabetic population [14–16]; however, the association between IR status or severity and cognitive function in the non-diabetic population remains unknown. Considering the large nondiabetic population, this association should be explored [17].

An association has been revealed between homeostasis model assessment insulin resistance (HOMA-IR) and cognitive function in the nondiabetic population [11, 18–21]. The triglyceride-glucose (TyG) index, which is computed from fasting blood glucose and triglyceride levels, is easier to record and cheaper compared to HOMA-IR [22]. It is a reliable marker to evaluate the IR status [23]. Compared to the HOMA-IR index, the TyG index was more strongly correlated with the hyperglycemic (Spearman coefficient of correlation: -0.64 [TyG] vs. -0.51 [HOMA-IR]) and the hyper-insulinemic euglycemic clamps (Pearson coefficient of correlation: -0.418 [TyG] vs. -0.324 [HOMA-IR]). Moreover, the hyper-insulinemic euglycemic clamp is the gold standard method for assessing IR [24, 25].

The TyG index is a valid surrogate marker of IR to assess the presence of nonalcoholic fatty liver disease (NAFLD) [26], and the severity of NAFLD, such as the presence of bladder cancer and coronary heart disease in NAFLD patients [27, 28]. A recent study provided Class II evidence that NAFLD was correlated with the development of nonvascular and vascular dementia [29]. The associations between the TyG index and cognitive performance, cognitive impairment, and dementia have been reported [14–16, 30–33]. However, all or some of the participants in these studies had diabetics. A cohort study reported the relationship between the fourth quartile of 5-years longitudinal changes in the TyG index and a decrease in global cognitive performance in men. Only the second quartile of the five-year change in the TyG index was strongly correlated with decreased cognitive performance in females [32]. Currently, the relationship between HOMA-IR and cognition in nondiabetic individuals is controversial [13, 34, 35]. Moreover, no study so far has reported a strong correlation between cognition and the TyG index in nondiabetic individuals. This study was the first to examine this relationship and perform subgroup analyses through using the National Health and Nutrition Examination Survey (NHANES).

## Methods

### Study population

Original data was obtained from the NHANES, which consists of a series of interviews and examinations of the civilian, noninstitutionalized US individuals organized through the Centers for Disease Control and Prevention (CDC). NHANES has been performed on a randomized and representative sample of U.S. populations every two years since 1999. NHANES uses a complex, stratified, and multistage design with sample weight to exactly estimate the prevalence of various diseases. NHANES aims to provide US de-identified health statistics that are made publicly available [36].

**Fig. 1:**
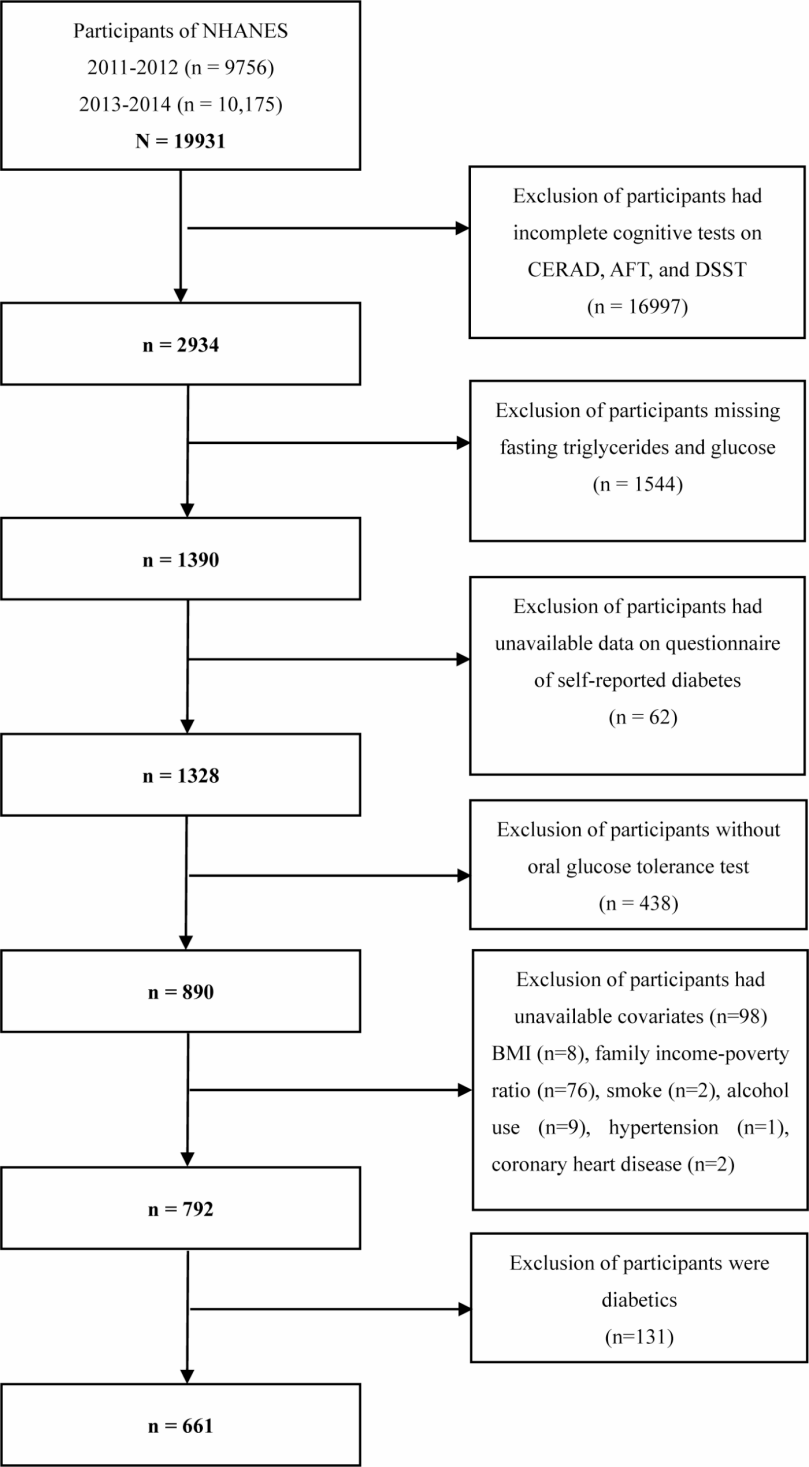
Selection of study population

This analysis included all participants from combining two 2-year cycles of NHANES 2011–2012 and 2013–2014 because only the two survey years measured three cognitive tests including Consortium to Establish a Registry for Alzheimer’s Disease battery for immediate word list learning and delayed word recall (also known as CERAD-WL and CERAD-DR), the Animal Fluency test (also known as AFT), and the Digit Symbol Substitution Test (also known as DSST). 19,931 participants were enrolled at first. This study excluded those individuals who had incomplete cognitive assessment tests on CERAD-WL, CERAD-DR, AFT, and DSST (n = 16,997). Then this study excluded those participants who missed the measurement of fasting triglycerides and fasting glucose (n = 1544) because their TyG index cannot be calculated. Next, this study excluded participants who had unavailable data on the questionnaire of self-reported diabetes (n = 62) and the measurement of the oral glucose tolerance test (OGTT) (n = 438). In addition, participants who had unavailable covariate data on body mass index (BMI) (n = 8), family income-poverty ratio (n = 76), smoke status (n = 2), alcohol use (n = 9), hypertension (n = 1) and coronary heart disease (n = 2) were excluded. This study defined diabetics as self-reported diabetics, serum fasting glucose ≥ 126 mg/dl, or 2-hour post-load glucose after OGTT ≥ 200 mg/dl. Those who were diabetics were excluded (n = 131). Finally, 661 participants were included in the analyses. The detailed selection process of the study population is presented in Fig. 1.

### Exposure variable and outcomes

The TyG index was computed through the previous formula: TyG = Ln [serum triglycerides (mg/dL) × serum glucose (mg/dL)/2] [37].

Several cognitive performance tests were introduced in the NHANES cycles 2011–2014. The NHANES 2011–2014 modules consist of CERAD-WL, CERAD-DR [38], AFT [39], and the DSST [40].

The CERAD test was conducted to evaluate the specific ability of new word learning, delayed recall, and recognition memories, consisting of three learning tests and an additional delayed recall test. Participants will be asked to read and recall ten words provided through NHANES staff in each test. It is worth noting that episodic memory or declarative memory is usually evaluated through the CERAD test. Each test’s score ranged from 0 to 10. The upper limit score for the total test (CERAD-WL and CERAD-DR) is 40. In this analysis, the CERAD test included the CERAD-WL cognitive performance test and the CERAD-DR cognitive performance test.

The AFT aims to examine categorical verbal fluency. Participants will be required to name animals in one minute through NHANES staff. A point will be given out for a successfully named animal in each asked.

The DSST was a cognitive performance test from the Wechsler Adult Intelligence Scale, depends on the speed of processing, the ability of sustained attention and memory in working of individuals, and is an experimental tool for understanding associative learning in humans [41]. Participants need to repeat the corresponding graphic symbol in one hundred and thirty-three boxes in two minutes. A point is given for each correct match with a total of 133 points. Currently, no formal standard of cutoff point was established for the CERAD test (CERAD-WL and CERAD-DR), AFT, and DSST tests to identify low cognitive function in participants. In accordance with previously published studies, this study defined the 25th quantile of each cognitive test score of each test as the cutoff point [42, 43].

### Covariates

Potential confounding factors included age, sex, race/ethnicity (White race, Black race, or other race), education levels (below high school, high school graduate, or above high school), the income-poverty ratio of family (< 1, ≥1), BMI levels (<25, 25–30, or ≥30), serum total cholesterol (TC) levels (mg/dl), smoking status (individuals who smoking ≥100 cigarettes during the whole life were defined as smokers), alcohol drinking (individuals who consuming ≥ 12 alcohol drinks every year was defined as alcohol users), physical activity (do any moderate-intensity sports ≥10 min continuously). Self-reported diseases in the interviews included hypertension, heart failure, coronary heart disease, and stroke.

### Statistical analysis

The complicated survey design was taken into consideration using specific sample weights following NHANES analytic standards [44]. All data for a cycle was collected in a single interview in this study. This research population’s weighted baseline information was represented as mean and standard deviation (SD) for any continuous variable and percentages for any categorical variable. The TyG index was further used to analyze both in continuous variables and quartiles. Weighted linear regression analyses were applied to compute β and 95% confidential interval (CI) to detect any potential association between the TyG index (continuous or quartiles) and three cognitive tests scores. Logistic regression analyses were applied to compute the odds ratio (OR) and CI to detect any potential association between the TyG index (continuous and quartiles) and low cognitive function. No covariates were adjusted for the crude model. Age and gender were adjusted in our Model 1; Model 2 was extra adjusted for BMI, race/ethnicity, educational level, family income-poverty ratio, serum TC levels, physical activity, smoking, alcohol drinking status, and self-reported diseases (history of hypertension, heart failure, coronary heart diseases, and stroke). Subgroup analyses were performed stratified through gender and smoking. IBM SPSS Statistics (version 24.0) and R software (version 4.2.2) were used for analyses. The *P* value < 0.05 was utilized as the statistical significance criterion.

## Results

### Characteristics of study population

661 non-diabetic individuals were contained in this study, 53.0% were females and 83.8% were non-Hispanic White race, with an average age of 68.62 ± 6.49 years. The mean value of the TyG index was 8.52 ± 0.55. A high prevalence of hypertension and overweight (BMI ≥ 25 kg/m^2^) was observed. The average score of the CERAD test, the AFT, and the DSST were 26.93, 18.82, and 54.70, respectively. The cutoff scores of low cognitive functions evaluated through the CERAD test, AFT, and DSST were 23, 15, and 43, respectively. Weighted baseline information of the study population based on the quartiles of the TyG index were presented in Table 1.

**Table 1:** Weighted baseline characteristics of participants

Characteristics	Overall (n = 661)	Q1 (n = 163)	Q2 (n = 172)	Q3 (n = 174)	Q4 (n = 152)	*P* value
Age, y	68.62 (6.49)	68.15 (6.59)	68.47 (6.75)	69.30 (6.57)	68.57 (6.04)	0.479
Female gender (%)	53.0	54.9	52.0	49.5	55.4	0.783
BMI, kg/m^2^ (%)						< 0.001
<25	31.4	47.4	35.2	27.0	16.3	
25–30	38.7	35.6	42.6	45.9	31.0	
≥30	29.8	17.0	22.2	27.0	52.7	
Ethnicity (%)						< 0.001
Non-Hispanic White	83.8	82.4	84.3	81.7	86.6	
Non-Hispanic Black	6.8	10.2	8.0	7.1	2.1	
Others	9.4	7.4	7.8	11.2	11.2	
Education (%)						0.122
Below high school	4.3	5.2	5.2	3.8	3.0	
High school graduate	7.5	2.8	9.3	10.6	7.5	
Above high school	88.2	92.0	85.5	85.6	89.6	
Income-poverty ratio	3.36 (1.50)	3.59 (1.48)	3.41 (1.50)	3.21 (1.60)	3.23 (1.39)	0.309
Smoking (%)	49.9	45.1	48.3	53.5	52.8	0.570
Alcohol drinking (%)	76.2	74.4	78.3	72.5	79.6	0.522
Physical activity (%)	48.8	56.7	47.4	48.6	42.7	0.236
Hypertension (%)	54.4	41.6	41.3	61.3	73.0	< 0.001
Heart failure (%)	6.1	4.4	2.4	5.3	12.2	0.017
Coronary heart disease (%)	7.2	7.1	7.7	7.1	6.8	0.988
Stroke (%)	5.2	9.3	3.8	2.8	4.7	0.066
TC, mg/dl	197.99 (39.41)	187.04 (33.64)	195.04 (37.73)	199.78 (41.25)	209.96 (41.28)	< 0.001
TyG index	8.52 (0.55)	7.85 (0.25)	8.31 (0.11)	8.67 (0.12)	9.24 (0.27)	< 0.001
CERAD test score	26.93 (6.20)	28.12 (6.54)	27.40 (5.53)	26.29 (6.35)	25.94 (6.15)	0.024
AFT score	18.82 (5.59)	19.67 (6.36)	18.99 (5.33)	18.20 (5.56)	18.42 (4.98)	0.332
DSST score	54.70 (16.67)	55.99 (17.64)	54.97 (16.01)	52.43 (17.67)	55.36 (15.21)	0.548

Abbreviations: The Consortium to Establish a Registry for Alzheimer’s Disease (CERAD); Digit Symbol Substitution Test (DSST); Animal Fluency test (AFT); TC: Total cholesterol

The CERAD test included CERAD-WL and CERAD-DR.

Continuous variables were shown in mean (SD) and categorical variables were shown in percentages

### Association between the TyG index and cognition function

After fully adjusting confounding factors, the fourth quartile of the TyG index was strongly correlated with the CERAD test [β: -2.45; 95% CI (-4.03, -0.96); *P* < 0.01]. No significant correlations were found between the TyG index and the AFT test or DSST test scores (Table 2).

**Table 2:** The association between TyG index and three cognitive test scores among nondiabetic older adults

	CERAD test score				Animal Fluency Test score				DSST score		
	Crude	Model 1	Model 2		Crude	Model 1	Model 2		Crude	Model 1	Model 2
TyG index (continuous)	-1.14 (-2.24, -0.03) ^*^	-1.02 (-2.10, 0.06)	-1.24 (-2.52, 0.03)		-0.71 (-1.77, 0.34)	-0.68 (-1.67, 0.32)	-1.03 (-2.16, 0.10)		-0.38 (-3.84, 3.07)	-0.07 (-3.07, 2.93)	-1.54 (-4.20, 1.11)
TyG index (quartiles)											
Q1	Reference				Reference				Reference		
Q2	-0.73 (-2.34, 0.89)	-0.54 (-1.88, 0.79)	-0.39 (-1.76, 0.97)		-0.68 (-2.52, 1.16)	-0.61 (-2.22, 1.00)	-0.65 (-2.38, 1.09)		-1.01 (-6.49, 4.45)	-0.02 (-0.11, 0.09)	-0.57 (-4.55, 3.41)
Q3	-1.84 (-3.56, -0.11) ^*^	-1.29 (-2.83, 0.25)	-1.15 (-2.81, 0.51)		-1.48 (-3.30, 0.35)	-1.22 (-2.89, 0.45)	-1.09 (-2.67, 0.48)		-3.56 (-9.41, 2.28)	0.03 (-0.09,0.15)	-1.97 (-6.57, 2.63)
Q4	-2.18 (-3.81, -0.55) ^*^	-2.04 (-3.53, -0.55) ^**^	-2.45 (-4.03, -0.96) ^**^		-1.25 (-3.04, 0.53)	-1.15 (-2.79, 0.49)	-1.54 (-3.27, 0.18)		-0.63 (-5.89, 4.64)	-0.08 (-0.19, 0.03)	-2.14 (-6.43, 2.15)

Abbreviations: The Consortium to Establish a Registry for Alzheimer’s Disease (CERAD); Digit Symbol Substitution Test (DSST)

Data are presented as β (95% confidence intervals). * *P* < 0.05. ** *P* < 0.01

Crude model adjusted for None

Model 1 adjusted for age and gender

Model 2 adjusted for age, gender, BMI, race/ethnicity, educational level, family income-poverty ratio, serum total cholesterol (mg/dl), physical activity, drinking status, smoking status, hypertension, heart failure, coronary heart disease, and stroke

The CERAD test included CERAD-WL and CERAD-DR.

### Association between the TyG index and cognition function

After fully adjusting confounding factors, the TyG index (continuous) was strongly correlated with low cognitive function evaluated through the CERAD test [OR: 1.61; 95% CI (1.01, 2.57); *P* < 0.05]. No associations were found between the TyG index as a continuous variable and low cognitive function evaluated through AFT and DSST tests.

Besides, compared to the 1st quartile of the TyG index, participants in the fourth quartile were significantly associated with low cognitive function evaluated through the CERAD test [OR: 2.62; 95% CI (1.31, 5.23); *P* < 0.05]. No associations were found between the TyG index as quartiles analysis and low cognitive function evaluated through AFT and DSST tests (Table 3).

**Table 3:** The association between TyG index and low cognitive function among nondiabetic older adults

	Low cognitive function assessed by CERAD test				Low cognitive function assessed by Animal Fluency Test				Low cognitive function assessed by DSST		
	Crude	Model 1	Model 2		Crude	Model 1	Model 2		Crude	Model 1	Model 2
TyG index (continuous)	1.43 (1.00, 2.04) ^*^	1.50 (0.99, 2.66)	1.61 (1.01, 2.57) ^*^		1.06 (0.68, 1.63)	1.05 (0.68, 1.63)	1.29 (0.71, 2.36)		0.84 (0.57, 1.24)	0.80 (0.52, 1.22)	0.88 (0.46, 1.67)
TyG index (quartiles)											
Q1	Reference				Reference				Reference		
Q2	1.11 (0.61, 2.00)	1.05 (0.58, 1.89)	0.95 (0.49, 1.82)		1.04 (0.60, 1.79)	1.01 (0.62, 1.66)	1.05 (0.52, 2.10)		0.97 (0.54, 1.37)	0.90 (0.48, 1.67)	0.81 (0.30, 2.16)
Q3	1.43 (0.77, 2.64)	1.27 (0.63, 2.55)	1.20 (0.57, 2.50)		1.30 (0.74, 2.28)	1.21 (0.71, 2.05)	1.32 (0.73, 2.37)		1.34 (0.68, 2.65)	1.18 (0.61, 2.29)	1.21 (0.51, 2.85)
Q4	2.02 (1.12, 3.66) ^*^	2.20 (1.14, 4.26) ^*^	2.62 (1.31, 5.23) ^*^		0.97 (0.49, 1.91)	0.94 (0.48, 1.83)	1.21 (0.49, 3.01)		0.68 (0.35, 1.32)	0.64 (0.32, 1.29)	0.76 (0.29, 1.99)

Abbreviations: The Consortium to Establish a Registry for Alzheimer’s Disease (CERAD); Digit Symbol Substitution Test (DSST)

Data are presented as OR (95% confidence intervals). * *P* < 0.05. ** *P* < 0.01

Crude model adjusted for None; Model 1 adjusted for age and gender; Model 2 adjusted for age, gender, BMI, race/ethnicity, educational level, family income-poverty

ratio, serum total cholesterol (mg/dl), physical activity, drinking status, smoking status, hypertension, heart failure, coronary heart disease, and stroke

The CERAD test included CERAD-WL and CERAD-DR.

Low cognitive function was defined the 25th quantile of each cognitive test score of each test as the cutoff point (CERAD: 23; AFT: 15; DSST: 43)

### Subgroup analyses

Subgroup analyses stratifying through gender and smoking were performed. The results of the subgroup analyses showed that females (OR_Q4 VS Q1_: 3.07; 95% CI (1.04, 9.05); *P* < 0.05) and smokers (OR _Q4 VS Q1_: 2.70; 95% CI (1.01, 7.26); *P* < 0.05) categories were related with a higher risk of low cognitive function, while no significant association was found in males and non-smokers categories (Table 4).

**Table 4:** Subgroup analyses

	CERAD test	Animal Fluency Test	DSST
Gender			
Female (continuous, n = 333)	1.74 (0.93, 3.26)	1.46 (0.74, 2.86)	0.87 (0.30, 2.53)
Q1	Reference		
Q2	0.78 (0.23, 2.65)	1.26 (0.37, 4.35)	0.40 (0.12, 1.38)
Q3	1.04 (0.36, 3.01)	0.85 (0.30, 2.43)	1.06 (0.36, 3.13)
Q4	3.07 (1.04, 9.05) *	1.33 (0.46, 3.84)	0.73 (0.16, 3.35)
Male (continuous, n = 328)	1.48 (0.69, 3.18)	1.17 (0.47, 2.92)	0.85 (0.32, 2.29)
Q1	Reference		
Q2	1.06 (0.38, 2.97)	0.80 (0.21, 3.01)	1.17 (0.24, 5.59)
Q3	1.30 (0.38, 4.48)	2.00 (0.65, 6.19)	1.23 (0.39, 3.92)
Q4	2.26 (0.73, 7.00)	1.13 (0.24, 5.45)	0.63 (0.14, 2.76)
Smoking			
Yes (continuous, n = 329)	1.77 (0.95, 3.27)	1.41 (0.66, 2.98)	1.06 (0.57, 1.98)
Q1	Reference		
Q2	0.98 (0.42, 2.26)	0.98 (0.34, 2.83)	1.44 (0.37, 5.62)
Q3	1.71 (0.53, 5.45)	1.52 (0.68, 3.39)	2.16 (0.86, 5.43)
Q4	2.70 (1.01, 7.26) *	1.71 (0.52, 5.64)	1.14 (0.46, 2.80)
No (continuous, n = 332)	1.45 (0.57, 3.69)	1.16 (0.48, 2.79)	0.55 (0.16, 1.90)
Q1	Reference		
Q2	1.09 (0.56, 2.10)	1.00 (0.36, 2.82)	0.35 (0.12, 1.02)
Q3	0.84 (0.28, 2.52)	0.91 (0.34, 2.45)	0.47 (0.17, 1.34)
Q4	2.86 (0.79, 10.34)	0.81 (0.19, 3.41)	0.38 (0.08, 1.73)

Abbreviations: The Consortium to Establish a Registry for Alzheimer’s Disease (CERAD); Digit Symbol Substitution Test (DSST)

Individuals who smoking ≥≥100 cigarettes during the whole life were defined as smokers

Data are presented as OR, 95% confidence intervals. * *P* < 0.05. ** *P* < 0.01

All analyses were based on model 2. The CERAD test included CERAD-WL and CERAD-DR.

## Discussion

This study included 661 US nondiabetic older adults and explored the correlation between the IR evaluated through the TyG index and low cognitive function evaluated through three cognitive tests in the NHANES 2011–2014. After fully adjusting for confounding factors that may influence cognitive function, the fourth quartile of the TyG index in participants was strongly correlated with low cognitive function evaluated through the CERAD test. No associations were determined between the IR evaluated through the TyG index and low cognitive function evaluated through the AFT and DSST tests. After stratified through gender and smoking, this study found that this relationship was significant in females and smokers subgroups. This present study indicated that examination and management of IR evaluated through the TyG index might be beneficial to improve cognitive function among nondiabetic older adults.

Previous studies on the correlation between cognitive function and IR evaluated through HOMA-IR in nondiabetic populations remained controversial. Some studies revealed that HOMA-IR was inversely related to cognitive performance among older adults [35, 45, 46], while other studies involved young adults or middle-aged women not [18, 19]. In a recent study using NHANES 2011–2014 data, they found the HOMA-IR index was not significantly related to the CERAD test, AFT, or DSST tests in older adults [47]. In these analyses, the fourth quartile of the TyG index in participants was strongly correlated with lower cognitive function evaluated through the CERAD test in women. This may be attributed to the selection of participants, the sensitivity of the HOMA-IR index compared with the TyG index, and whether the used quantiles analyses were. Interestingly, a recent cohort study has shown the five-year alteration of the TyG index and cognitive function in China [32]. They reported that increased TyG index was strongly correlated with cognitive decline in men and the second quartile of the longitudinal change of TyG index was significantly correlated with decreased cognitive performance evaluated through the CERAD test in females. Females were more likely to suffer from cognitive impairment than males [3]. A prospective Danish study revealed that IR increased cognitive impairment risk in women [48]. Ekblad et al. demonstrated that higher HOMA-IR was significantly related to poorer verbal fluency in females [49]. This could be attributed to the decrease in estrogen and treatment with estradiol was considered to improve cognitive function [50]. The relationship between the IR evaluated through the TyG index and cognitive function evaluated through the CERAD test was found significant in two large samples in China and the US separately, future studies may need to validate this relationship and find underlying mechanisms. Besides, a study reported the correlation between TyG index and dementia, and they found after stratifying through age, gender, smoking, and alcohol use, the significant association did not change [30]. In this study, stratifying analyses through smoking showed that this relationship was more significant among self-reported smokers. Tobacco smoke exposure can also impair brain insulin signaling (63), and is also a risk factor for NAFLD (64). As for those who did not smoke, they may have health habits that were protective of cognitive function.

The results could be explained by several possible mechanisms. Insulin serves to regulate the function of learning and memory in the brain [51]. Brain IR can impair the function of insulin, leading to poor learning and memory [52, 53], and peripheral IR has been found to reduce insulin transport across the blood-brain barrier [54]. In addition, hyperinsulinemia may dysregulate central insulin signaling by reducing the insulin transport across the blood-brain barrier [55–57]. IR is a characteristic metabolic disorder that coexists with hyperinsulinemia, long-term exposure of brain neurons to elevated insulin levels also causes neurodegeneration and permanent memory impairment [58]. Besides, IR could decrease the regional metabolism of cerebral glucose, which might predict worse memory function in individuals [59]. Moreover, plenty of evidence has shown a higher prevalence of cognitive impairment in patients with depression [60], and two recent studies revealed that the TyG index was associated with the presence of depression [61, 62]. According to the proposed mechanism, this study demonstrated that IR has a substantial inverse association with cognitive performance.

### Study strengths and limitations

This present study provided clinical evidence of the positive association between the TyG index and low cognitive function. In addition, the results were relatively convincing because of using a large, US representative cohort with rigorous quality control. However, some limitations in this present study that cannot be ignored. First, due to the cross-sectional design, causality could not be determined. Second, although NHANES uses multiple tests to assess the cognitive function of older adults, this still may not represent cognitive function comprehensively. Third, some confounding factors were not completely adjusted, such as the use of medicine.

## Conclusions

A high TyG index was significantly associated with low cognitive function evaluated through the CERAD test among US nondiabetic older adults. No relationship was detected between the TyG index and low cognitive function evaluated through AFT and DSST tests. This study indicated that the TyG index may be a useful predictor of low cognitive function. The examination and management of the TyG index might be beneficial to improve cognitive function among nondiabetic older adults. More high-quality multi-center studies are needed to testify to these findings, which might be useful to guide risk prediction and primary prevention of low cognitive function in nondiabetic older adults.

## Data Availability

The dataset supporting the conclusions of this article is available in the https://wwwn.cdc.gov/nchs/nhanes/Default.aspx.

## Abbreviations

IR: Insulin resistance
TyG: Triglyceride glucose
CERAD: Consortium to Establish a Registry for Alzheimer’s Disease Battery
CERAD-WL: Consortium to Establish a Registry for Alzheimer’s Disease Battery for immediate word list learning
CERAD-DR: Consortium to Establish a Registry for Alzheimer’s Disease Battery for delayed recall
AFT: Animal Fluency Test
DSST: Digit Symbol Substitution Test
NAFLD: Nonalcoholic fatty liver disease
OR: Odds ratio
CI: Confidential interval
SD: Standard deviation
HOMA-IR: Homeostasis model assessment insulin resistance
OGTT: Oral glucose tolerance test
BMI: Body mass index
NHANES: National Health and Nutrition Examination Survey
TC: Total cholesterol
